# Development of a fixed-bed anammox reactor with high treatment potential

**DOI:** 10.1007/s10532-012-9561-x

**Published:** 2012-06-10

**Authors:** Hiroyuki Okamoto, Kimito Kawamura, Takashi Nishiyama, Takao Fujii, Kenji Furukawa

**Affiliations:** 1 Research & Development Laboratories for Sustainable Value Creation, Asahi Group Holdings, Ltd., 1-1-21 Midori, Moriya, Ibaraki 302-0106 Japan; 2 Department of Applied Life Science, Sojo University, 22-1, Ikeda 4-Chome, Kumamoto-shi, Kumamoto 860-0082 Japan; 3 Graduate School of Science and Technology, Kumamoto University, 2-39-1, Kurokami, Kumamoto 860-8555 Japan; 4 318 Nishikawahara-Cho, Moriyama-ku, Nagoya, 463-0089 Japan

**Keywords:** Anammox, Charcoal, Biomass carriers, Spent grains, Nitrogen removal

## Abstract

A plug-flow type anaerobic ammonium oxidation (anammox) reactor was developed using malt ceramics (MC) produced from carbonized spent grains as the biomass carriers for anammox sludge. Partial nitrified effluent of the filtrate from the sludge dehydrator of a brewery company was used as influent to a 20 L anammox reactor using MC. An average volumetric nitrogen removal rate (VNR) of 8.78 kg-N/m^3^/day was maintained stably for 76 days with 1 h of HRT. In a larger anammox reactor (400 L), an average VNR of 4.84 kg-N/m^3^/day could be maintained for 86 days during the treatment of low strength synthetic inorganic wastewater. As a result of bacterial community analysis for the 20 L anammox reactor, Asahi BRW1, probably originating from the wastewater collected at Asahi Breweries, was detected as the dominant anammox bacterium. These anammox reactors were characterized by a high NH_4_-N removal capacity for low strength wastewater with a short hydraulic retention time.

## Introduction

Nitrogen and phosphorus compounds containing in food, chemical fertilizers, raw materials for industry and fuel are discharged in wastewater when these materials are used and it is important to reduce their levels in wastewater to prevent eutrophication. Ammonium nitrogen (NH_4_-N) can be removed from wastewater in a multi-step process using ammonium oxidizing bacteria (AOB) and nitrite oxidizing bacteria (NOB), which convert NH_4_-N to nitrate nitrogen (NO_3_-N). NO_3_-N is then subsequently reduced to nitrogen gas by biological denitrification. Recently, a new nitrogen removal process using autotrophic anaerobic ammonium oxidation (anammox) bacteria has attracted much attention. Anammox bacteria can convert NH_4_-N and nitrite nitrogen (NO_2_-N) to nitrogen gas directly under anaerobic conditions, as shown in Eq. 1.

1 $$ 1.0{\text{NH}}_{4}^{+} + 1.32{\text{NO}}_{2}^{-} + 0.066{\text{HCO}}_{3}^{-} + 0.13{\text{H}}^{+} \to 1.02{\text{N}}_{2} + 0.26{\text{NO}}_{3}^{-} + 0.066{\text{CH}}_{2} {\text{O}}_{0.5} {\text{N}}_{0.15} + 2.03{\text{H}}_{2} {\text{O }} $$

The anammox process can reduce excess sludge production, energy requirements and the footprints of wastewater treatment plants. Therefore, the anammox process can reduce greenhouse gas production from wastewater treatment. However, the anammox process is difficult to apply practically owing to the extremely slow growth rate of anammox bacteria (doubling time 11 days) (Van Dongen et al. 2001). It is also difficult to maintain autotrophic anammox bacteria in the presence of organic compounds (Chamchoi et al. 2008). Overcoming this limitation, the first anammox plant was constructed in the Netherlands (Van der Star et al. 2007). Technology for mass cultivation of anammox bacteria using nonwoven polyester as a biomass carrier has also been developed in Japan (Rouse et al. 2003) and can be easily installed in an anammox plant. Breweries discharge several thousands tons of nitrogen-containing wastewater every day. Application of the anammox process to wastewater treatment for breweries would be an effective way to reduce the cost of wastewater treatment in this industry. Our group has been studying the application of anammox process with high-speed nitrogen removal to brewery wastewater. Because of the low growth rate of anammox bacteria, it is important to prevent their washout from the reactor. Several methods have been developed to retain a high density of anammox bacteria in the reactor. One method uses granules from an upflow anaerobic sludge blanket (UASB) reactor as a carrier for anammox bacteria (Imajo et al. 2005; Tokutomi 2006), while another uses immobilized anammox pellets (Isaka 2004).

The NH_4_-N concentration in brewery wastewater is in the range of hundreds of milligrams per liter, which is relatively low compared with other types of wastewater. Application of the anammox process to a wastewater containing low nitrogen concentration requires operation under a short hydraulic retention time (HRT). Granules from a UASB reactor used as biomass carriers (Tokutomi and Yasui 2003) will be easily washed out from the reactor under a short HRT and this would make stable wastewater treatment difficult. When polyethylene glycol is used for whole cell entrapment, it is necessary to use agitation, requiring electrical energy in the reactor. Plug flow reactors are smaller than other reactors, according to reaction engineering theory (Komiyama 1995), and this would provide energy savings during operation of the anammox process. A maximum volumetric nitrogen removal rate (VNR) of 5.36 kg-N/m^3^/day was obtained through the continuous treatment of synthetic inorganic wastewater using a plug flow anammox reactor with a total volume of 1.62 L (Hoa et al. 2006; Luong et al. 2007). The biomass carrier used in this reactor was malt ceramics (MC), which is a type of charcoal made from used dry spent grains (Okamoto et al. 2002). On the other hand, Imajo et al. obtained VNRs of 1.55, 2.38, and 2.87 kg-N/m^3^/day using nonwoven polyester, plastic pipe, and granules from a UASB reactor, respectively, as biomass carriers for the anammox sludge (Imajo et al. 2005). These results illustrate that MC is an excellent biomass carrier for anammox sludge. Our group has investigated the treatment of brewery wastewater containing organic compounds using a plug flow anammox reactor (total volume 11 L) filled with MC as biomass carriers. The maximum VNR obtained with this system was 4.17 kg-N/m^3^/day with a HRT of 42 min and an influent C/N ratio of 0.196 (Okamoto et al. 2009a, b). We are now trying to scale up this anammox reactor for aiming practical applications. Here, we report the results for two plug-flow anammox reactors with total volumes of 20 and 400 L.

## Materials and methods

### Analysis of wastewater samples

All wastewater samples were left to stabilize for more than 10 min and then the water quality was measured, except for the TOC. Influent and effluent water qualities were analyzed as follows. The NH_4_-N concentration was detected using an ammonia sensor (Ti-9001 Ion Meter, Toko Kagaku Inc., Tokyo Japan: Seven Multi, Mettler-Toledo International Inc., Columbus, OH). NO_2_-N and NO_3_-N concentrations were measured using a portable analyzer (TNP-10, DKK-TOA Corp., Tokyo, Japan). The pH was measured using a portable pH sensor (D-55, Horiba, Ltd., Kyoto, Japan: HM-30R, DKK-TOA Corp.). For the measurement of TOC, the samples were centrifuged at 3,000 rpm and the supernatant was analyzed using a TOC analyzer (TOC-V, Shimadzu Corp., Kyoto, Japan). The bacterial community in biomass was analyzed as follows. DNA was extracted from each sludge sample by using ISOIL kit (Nippon gene, Osaka, Japan). Extracted DNA was subjected to PCR amplification by using the primer pair 16S6F and 1492r. Oligonucleotide sequences of these primers were 16S6F (Tchelet et al. 1999): 5′-GGAGAGTTAGATCTTGGCTCAG-3′, 1492r (Lane 1991): 5′-GGTTACCTTGTTACGACT-3′. The amplified DNA products were cloned and sequenced.

### Experiment with 20 L anammox reactor

#### Wastewater

Many types of wastewater are discharged from brewery processes. In the present study, wastewater with a high concentration of NH_4_-N was collected from Asahi Breweries (Tokyo, Japan). About 4.9 % of the total volume of wastewater produced by this brewery is filtrate from a sludge dehydrator. However, the amount of nitrogen contained in this filtrate is reached to about 14.4 % of the total amount of nitrogen discharged (as estimated in 2009). Of the influent nitrogen, about 80 % of the total nitrogen is NH_4_-N. Consequently, this filtrate would be a potential target for the anammox treatment. In filtrate, NH_4_-N and TOC concentrations were about 88–432 and 17–689 mg/L, alkalinity concentrations was about 900–1710 mg/L as CaCO_3_, pH was about 7.2–8.3.

Pre-treatment is required to remove organic compounds and SS in the filtrate. We have developed a pretreatment process to remove these SS and organic compounds and to convert about 50 % of the NH_4_-N in the filtrate to NO_2_-N (partial nitritation) (Okamoto et al. 2009a, b).

In this study, pretreated filtrate was used as the influent to the anammox reactor. Synthetic inorganic wastewater was prepared using ammonium sulfate and sodium nitrite and tap water was added to the pretreated filtrate to increase the influent nitrogen concentration. Influent NH_4_-N and NO_2_-N concentrations were increased by 50 mg/L until day 218 (Run 1). From day 219 (Run 2), influent NH_4_-N and NO_2_-N concentrations were increased by 100 mg/L. Ferrous sulfate heptahydrate was added to the influent to increase the iron concentration by 3.6 mg/L and ethylene diamine tetra-acetic acid was added to the influent at 10 mg/L to prevent iron precipitation.

#### Reactor

**Fig. 1:**
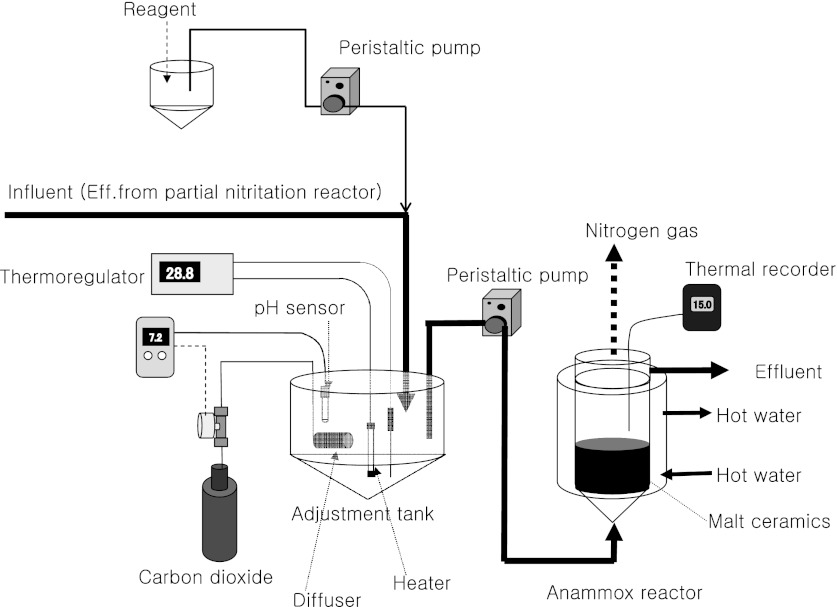
Treatment flow diagram for the 20 L anammox reactor

The 20 L anammox reactor (effective volume 19.2 L) was cylindroconical and filled with 4,000 g of MCP (about 8 L), which were MC particles with diameter less than 10 mm. Effluent from another anammox reactor was supplied to the 20 L reactor to inoculate it with anammox bacteria and then influent was supplied to the reactor for anammox treatment. The influent was supplied from an adjustment tank (effective volume 100 L) to the bottom of the reactor by a roller pump (RP-1000, Tokyo Rika Kikai Co, Ltd., Tokyo, Japan). The temperature of the adjustment tank was maintained at 25–35 °C by a heater and temperature controller. The temperature in the reactor was controlled at about 30 °C using a water jacket. The pH was automatically adjusted to below 7.5 using carbon dioxide and a pH sensor and controller (NPH-690D, Nissin Rika Co, Ltd., Tokyo, Japan) attached to the adjustment tank. A thermal data logger (TR-71U, T&D Corporation, Matsumoto, Japan) was used to record the temperature from 8:30 a.m. to 9:30 a.m. each day. The experimental setup and the treatment process are illustrated in Fig. 1.

### Experiment with 400 L anammox reactor

#### Wastewater

Synthetic inorganic wastewater mainly composed of ammonium sulfate, sodium nitrite, potassium dihydrogen phosphate and sodium bicarbonate, was used as the influent to the 400 L anammox reactor. The characteristics of the synthetic inorganic wastewater were as follows. NH_4_-N and NO_2_-N concentrations were about 57–212 and 62–265 mg/L, T-P and TOC concentrations were about 8–35 and 0–14 mg/L, alkalinity concentrations was about 180–260 mg/L as CaCO_3_, pH was about 6.9–7.9.

Influent NH_4_-N and NO_2_-N concentrations were gradually increased to 200 and 220 mg/L, respectively. Ferrous sulfate heptahydrate was added to the influent to increase the Fe concentration by 3.6 mg/L, and EDTA (10 mg/L) was added to prevent Fe precipitation. The quality of tap water used for preparation of synthetic inorganic wastewater was measured using inductively coupled plasma (ICP) and the concentrations of zinc, manganese and nickel were then adjusted to be equal to those in the pretreated brewery wastewater by addition of reagents to the tap water.

#### Reactor

The 400 L anammox reactor had an effective volume of 416 L and was cylindroconical with an acrylic resin cylinder and stainless steel conical section. A jacket filled with hot water around the reactor was used to maintain the temperature. The reactor was similar to the 20 L anammox reactor in configuration with the exception that equipped simplified gas solid separator and was filled with MCP and MCL inoculated with anammox bacteria. The MCL was MC particles under 15 mm in diameter. The synthetic inorganic wastewater was supplied to the bottom of reactor by a screw pump (Mono-screw pump: Iwaki Co. Ltd., Tokyo, Japan). Influent NH_4_-N and NO_2_-N concentrations were set to below 100 mg/L. Influent flow rate was increased gradually while maintaining effluent NO_2_-N concentrations below 20 mg/L. After reaching an influent flow rate of 4.2 L/min, influent NH_4_-N and NO_2_-N concentrations were increased gradually to their maximum values. The temperatures of the reactor and the synthetic inorganic wastewater were initially maintained at 35 °C. After reaching the set flow rate and the maximum nitrogen concentrations, the reactor temperature was decreased to 30 °C, and then 25 °C. Initially, until day 58, the synthetic inorganic wastewater was prepared manually but was prepared automatically thereafter. The pH of the synthetic inorganic wastewater was controlled at about 7.0 by addition of 50 g/L of aqueous sodium bicarbonate.

## Results and discussion

### Experiments with the 20 L anammox reactor

**Fig. 2:**
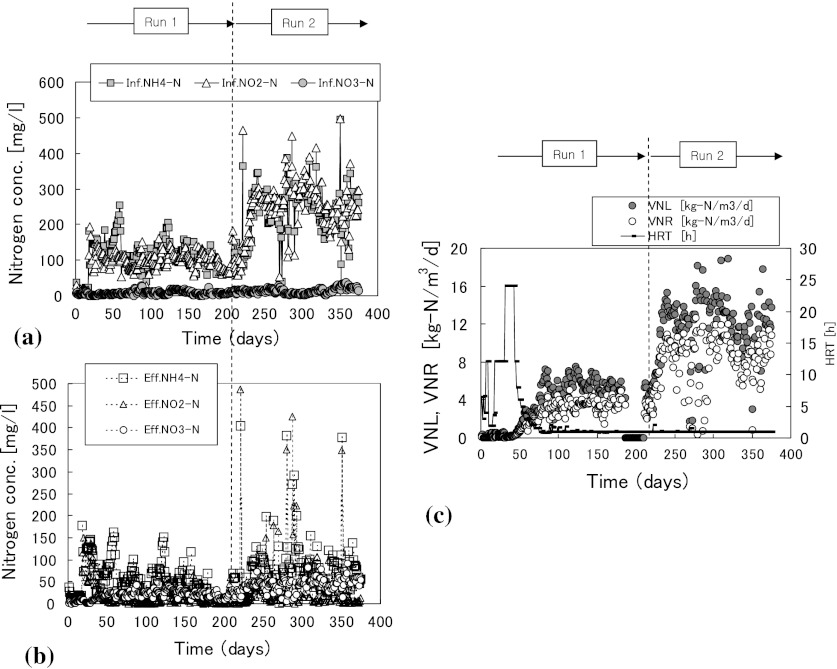
Daily changes in treatment conditions for the 20 L anammox reactor.** a** Influent.** b** Effluent.** c** VNL,VNR and HRT

Figure 2a, b shows the daily changes in pretreated influent and effluent nitrogen concentrations for the 20 L anammox reactor. The average, range, and standard deviation for NH_4_-N, NO_2_-N and NO_3_-N concentrations, TOC concentrations and pH are shown in Table 1. Figure 2c shows the daily changes in VNL, VNR and HRT. Here, total nitrogen was defined as the sum of nitrogen from NH_4_-N, NO_2_-N, and NO_3_-N.

**Table 1:** Treatment results for the 20 and 400 L anammox reactors

Item	Unit	Influent			Effluent		
		Average	Range	SD^a^	Average	Range	SD
20 L reactor, Run 1							
NH_4_-N	mg/L of N	120	39–254	35.7	57	0–177	38.4
NO_2_-N	mg/L of N	100	28–193	25.0	18	0–150	26.6
NO_3_-N	mg/L of N	8	0–29	5.3	21	1–46	8.4
TOC	mg/L of C		**–**	**–**	**–**	**–**	**–**
pH	–	7.27	6.6–7.8	0.26	7.62	6.9–8.4	0.33
20 L reactor, Run 2							
NH_4_-N	mg/L of N	237	31–495	69.3	79	1–404	64.2
NO_2_-N	mg/L of N	252	20–498	75.0	37	1–486	74.0
NO_3_-N	mg/L of N	14	2–37	7.8	45	6–92	13.7
TOC	mg/L of C	27	20–35	3.9	26	20–34	3.4
pH	–	7.21	6.8–7.9	0.24	7.85	6.9–8.7	0.38
400 L reactor, Run IV							
NH_4_-N	mg/L of N	185	154–205	11.5	29	15–77	12.1
NO_2_-N	mg/L of N	215	196–237	7.2	6	0–66	10.4
NO_3_-N	mg/L of N	**–**	**–**	**–**	32	24–38	2.4
TOC	mg/L of C	1.2	0.5–2.5	0.5	2.9	1.2–5.2	1.0
pH	–	7.22	7.1–7.6	0.09	8.50	8.2–8.8	0.09
Temp.	°C	**–**	**–**	**–**	31.9	25.0–36.0	2.4
400 L reactor, Run V							
NH_4_-N	mg/L of N	203	183–212	7.0	43	37–54	4.2
NO_2_-N	g/L of N	212	199–222	6.5	9	4–28	4.6
NO_3_-N	mg/L of N	**–**	**–**	**–**	31	27–34	1.9
TOC	mg/L of C	1.2	0.9–1.5	0.3	3.2	1.8–4.1	1.0
pH	–	7.28	7.2–7.4	0.03	8.48	8.3–8.6	0.07
Temp.	°C	**–**	**–**	**–**	29.6	27.0–31.0	0.8

*SD* standard deviation

There was a maximum difference of 200 mg/L in the influent NH_4_-N and NO_2_-N concentrations but there were no differences between effluent NH_4_-N and NO_2_-N concentrations during Runs 1 and 2 (Fig. 2a, b). These results show that the anammox process had sufficient buffering capacity to cope with the increase in the nitrogen load from Run 1 to Run 2. After attachment of anammox sludge on MC, influent NH_4_-N and NO_2_-N concentrations were set to 100–120 mg/L in Run 1 and 230–250 mg/L in Run 2.

In all cases, the sudden increase in effluent NO_2_-N concentration (Fig. 2b) or sudden decrease in VNR (Fig. 2c) were failures in preparation of the influent. Under proper management of these inhibiting factors, the nitrogen removal efficiencies were maintained to about 60–80 %.

The HRT was set to 12 h at the beginning of Run 1 but was changed to 24 h later to decrease the effluent NO_2_-N concentrations below the inhibition level of 20 mg/L. Effluent NO_2_-N concentrations could be maintained below 20 mg/L until day 43 and then the influent flow rate was increased gradually to increase the VNL.

On day 86, the VLR was increased above 4.0 kg-N/m^3^/day and reached a maximum of 4.45 kg-N/m^3^/day. After day 86, the HRT was fixed at 1.0 h and the average VNR was maintained at 3.47 kg-N/m^3^/day. After day 219 (Run 2), the quantity of supplemented chemicals to the influent was increased to increase VNR and VNL (Fig. 2c). The average VNR was 8.78 kg-N/m^3^/day from day 300 to 375.

**Fig. 3:**
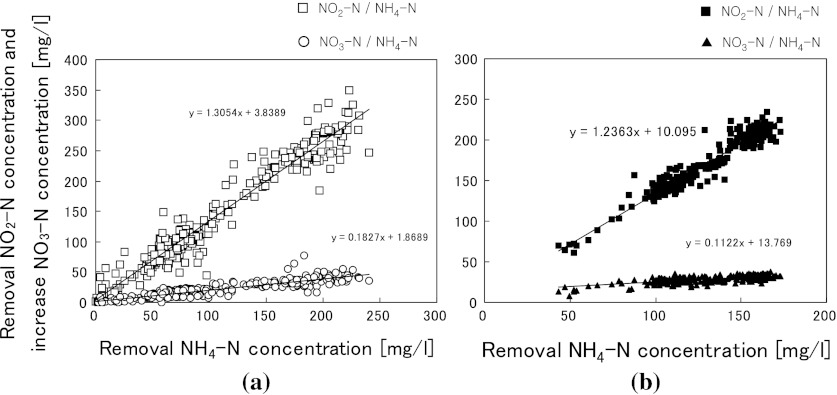
Reaction ratios for each anammox reactor. **a** 20 L anammox reactor. **b** 400 L anammox reactor

**Fig. 4:**
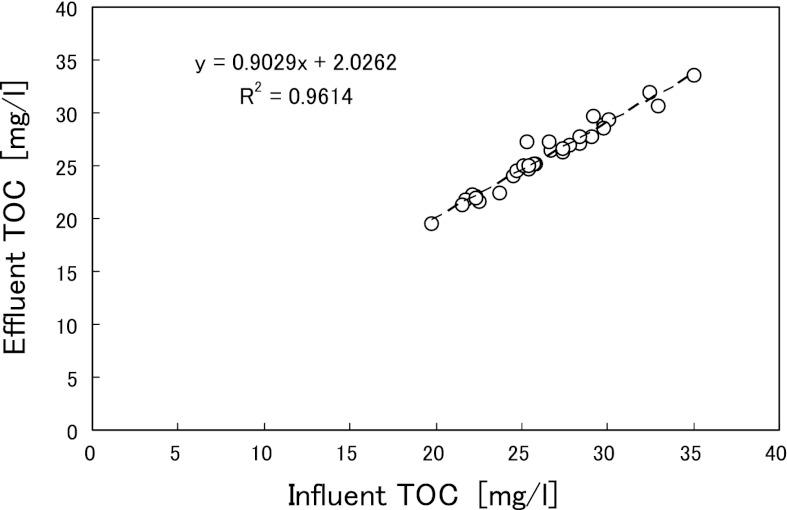
Relationship between influent TOC and effluent TOC for the 20 L anammox reactor

Figure 3 shows the relationship for differences in the nitrogen concentrations between the influent and effluent. The reaction ratios of NO_2_-N/NH_4_-N and NO_3_-N/NH_4_-N were calculated from the slopes of regression lines to be 1.31 and 0.18, respectively (Fig. 3). TOC concentrations were analyzed from day 253 (during Run 2). Figure 4 shows the relationship between influent and effluent TOC concentrations, for which there were no large differences (Table 1). These results suggest that removal of influent organic compounds was low during anammox treatment, with a removal rate of only about 10 %. At day 156 (during Run 1), anammox sludge samples were taken with biomass carrier and its bacterial community was analyzed. Table 2 shows the results of a homology search for the 37 clones. In the biomass sample from the 20 L anammox reactor, the highest existing ratio was obtained for the bacterium whose 16S rRNA gene sequence had 99 % identity with that of an anammox bacterium Asahi BRW1 (AB456583).

**Table 2:** Bacterial community analysis for sludge

In the 20 L anammox reactor				
Phylum (Class)	Best hit sequence in the nr-database	Identity [%]	Clone Number	Accession No.
Proteobacteria (β)	Uncultured bacterium clone KIST-JJY030	99	3	EF654699
	Uncultured bacterium clone: A	99		AB194898
	Uncultured bacterium clone 2.56	99	1	JN256103
	*Azonexus hydrophilus* strain IMCC1716	95		DQ664239
	Uncultured bacterium clone Q7334-HYSA	89	1	JN391835
	Uncultured Betaproteobacteria bacterium clone QEDN3AC09	89		CU927281
Planctomycetes	Uncultured bacterium clone: Asahi BRW1*	99	23	AB456583
	*Candidatus* Brocadia sp. clone 40*	99		AM285341
Acidobacteria	Uncultured bacterium clone Dok32	99	2	FJ710751
	Uncultured *Acidobacterium* sp. clone sw-xj91	95		GQ302572
Bacteroidetes	Uncultured bacterium clone GZKB119	95	1	AJ853611
	Uncultured Cytophagales bacterium clone TDNP_Wbc97_191_1_71	91		FJ517039
Chlorobi	Uncultured bacterium clone Dok61	99	2	FJ710780
	Uncultured Bacteroidetes/Chlorobi group bacterium clone D25_47	98		EU266920
Chloroflexi	Uncultured bacterium clone B57	97	1	HQ640553
	Uncultured Caldilineaceae bacterium clone T9222f9	91		HM447656
	Uncultured Chloroflexi bacterium clone: HUY-A12	97	1	AB638620
	Uncultured bacterium clone NIT-EN-19	96		HQ843697
	Uncultured Chloroflexi bacterium clone Alchichica_AQ1_1_1B_10	91	1	JN825477
	Uncultured bacterium clone 655947	90		DQ404819
	Uncultured Chloroflexi bacterium clone: HUY-B09	92	1	AB638622
	Uncultured bacterium clone KIST-JJY024	90		EF594056
	Total		37	

The seed for the 400 L anammox reactor				
Phylum (Class)	Best hit sequence in the nr-database	Identity [%]	Clone Number	Accession No.
Proteobacteria (β)	Uncultured bacterium clone KIST-JJY030	93-99	10	EF654699
	Uncultured bacterium clone: A	93-99		AB194898
Planctomycetes	Uncultured bacterium clone: Asahi BRW1*	94-99	5	AB456583
	*Candidatus* Brocadia sp. clone 40*	94-98		AM285341
	Uncultured bacterium clone: Asahi BRW2*	98-100	34	AB524902
	Planctomycete KSU-1*	93-94		AB057453
	Uncultured bacterium clone: A1*	98	1	AB462402
	*Candidatus* Brocadia sinica*	98		AB565477
	Uncultured bacterium clone Ge03	98	1	FJ710653
	Uncultured Planctomycetales bacterium clone T13J-B80	90		JN860385
	Uncultured bacterium clone ana_SBR_JJY_44	95	1	FJ577893
	Uncultured planctomycete clone 5GA_Pla_HKP_08	95		GQ356155
Chloroflexi	Uncultured bacterium clone KIST-JJY023	98	1	EF594055
	Uncultured bacterium clone Ge28	97		FJ710678
	Total		53	

In the partial-nitritation reactor (Okamoto et al. 2010) (Re-analysis)				
Phylum (Class)	Best hit sequence in the nr-database	Identity [%]	Clone Number	Accession No.
proteobacteria (β)	Uncultured bacterium clone KIST-JJY028	99	1	EF654697
	Uncultured Burkholderiales bacterium clone REV_R1PII_1A	96		FJ933489
	*Nitrosomonas* sp. ENI-11.1	93	1	AB079053
	*Nitrosomonas europaea* strain ATCC 19718	93		AL954747
Proteobacteria (δ)	Uncultured bacterium clone 080624-Aspo-Fracture-Biofilm-G1-10	92	1	FJ867344
	Uncultured delta proteobacterium clone 49S1_2B_46	89		DQ837252
Planctomycetes	Uncultured bacterium clone: Asahi BRW1*	99-100	26	AB456583
	*Candidatus* Brocadia sp. clone 40*	97-99		AM285341
	Uncultured bacterium clone ana_SBR_JJY_44	90	1	FJ577893
	Uncultured planctomycete clone 5GA_Pla_HKP_08	90		GQ356155
Acidobacteria	Uncultured bacterium clone Dok32	94-99	4	FJ710751
	Uncultured *Acidobacterium* sp. clone sw-xj91	92-96		GQ302572
	Uncultured bacterium clone KIST-JJY008	99	1	EF584528
	Uncultured Acidobacteriaceae bacterium clone EfT107_A12	96		GU201543
Actinobacteria	*Sphaerobacter thermophilus* DSM 20745	91	1	CP001823
	Uncultured bacterium clone D-16S-15	90		EU603380
Bacteroidetes	Uncultured bacterium clone SBRAC63	99	1	HQ158649
	Uncultured Saprospiraceae bacterium clone Epr33	95		EU177727
Chlorobi	Uncultured bacterium clone Dok23	99	4	FJ710742
	Uncultured *Ignavibacterium* sp. clone S122	96		JN217054
Chloroflexi	Uncultured Chloroflexi bacterium clone Z4MB91	97	1	FJ484890
	Uncultured bacterium clone 62	97		DQ413121
	Uncultured Chloroflexi bacterium clone CSBC1C05	97	1	GU126977
	Uncultured bacterium clone 24	96		FJ437973
Gemmatimonadetes	Uncultured bacterium clone LaP15L54	97	1	EF667669
	Uncultured Gemmatimonadetes bacterium clone Skagen138	95		DQ640715
candidate division OP10	Uncultured bacterium clone Ge64	99	2	FJ710714
	Uncultured candidate division OP10 bacterium clone HAVOmat40	90-92		EF032775
	Total		46	

Asterisks indicate anammox bacteria

### Experiments with the 400 L anammox reactor

**Fig. 5:**
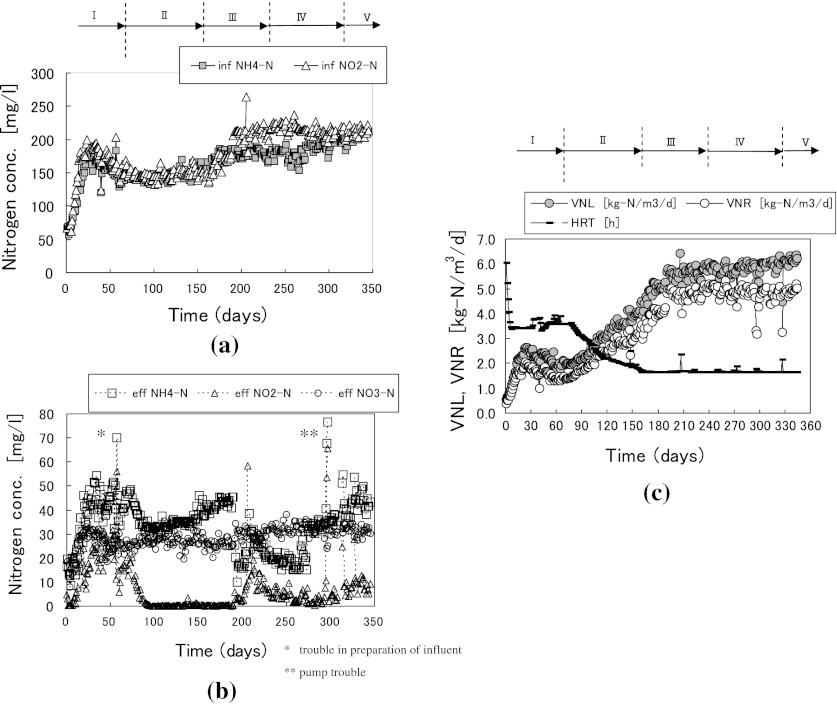
Daily changes in treatment conditions for the 400 L anammox reactor.** a** Influent.** b** Effluent.** c** VNL,VNR and HRT

As shown in Table 3, the 400 L anammox reactor experiment was divided into five runs (Runs I–V). The changes in the influent nitrogen concentrations (NH_4_-N and NO_2_-N) are shown in Fig. 5a and the daily changes in effluent nitrogen concentrations and operational troubles are shown in Fig. 5b. Treatment results for Runs IV and V are shown in Table 1. The daily changes in VNL and VNR and the HRT are shown in Fig. 5c.

**Table 3:** Operational tactics for the 400 L anammox reactor

Period	Plan to operate anammox reactor	Target of control value			Term (days)
		Influent NH_4_-N and NO_2_-N concentrations (mg/L)	Flow rate (L/min)	Temp. (°C)	
Run I	Some improvements were made to the anammox reactor	From 70 to 150	From 1.15 to 2.00	35	0–71
Run II	First increasing stage in VNR As a result of improvement, VNRs were increased Flow rate was slowly increased without increasing influent nitrogen concentrations	150	From 2.00 to 4.20	35	72–160
Run III	Second increasing stage in VNR Influent concentration was slowly increased without increasing influent flow rate	From 150 to 200 or 220	4.20	35	161–231
Run IV	Stable operational stage Treatment stability was evaluated under constant VNL	200 (NH_4_-N) 220 (NO_2_-N)	4.20	35	232–317
Run V	Cooling stage Reactor temperatures were decreased without reducing VNL	200 (NH_4_-N) 220 (NO_2_-N)	4.20	35–30	318–345

The VNR peaked on day 24, up to day 71 (Run I) and some improvements were then made to the system. From day 72 on (Run II), the VNR was increased as shown in Fig. 5c. Until day 160, the VNR was increased by increasing the influent flow rate, while influent NH_4_-N and NO_2_-N concentrations were fixed at 150 mg/L. On day 160, the influent flow rate reached 4.2 L/min (HRT = 1.7 h), which was the maximum capacity of the feed pump.

After day 161 (Run III), the VNL was increased by increasing the influent nitrogen concentration, while the influent flow rate was fixed at 4.2 L/min. On day 225, the operational conditions reached their target values (VNR > 5.0 kg-N/m^3^/day, HRT < 2.0 h).

After day 232 (Run IV), the reactor was operated under constant operational conditions with no further changes in the influent flow rate or influent nitrogen concentration. The maintainability of the anammox reactor under the maximum nitrogen removal rate was evaluated through this operation. On day 296 (Run IV), a number of operational troubles occurred but the VNR and removal efficiency were recovered within only two days when the operational conditions were properly adjusted. During Run IV from mid-October to early January, the ambient room temperature was decreased. The reactor temperature was decreased from 35 to 31 °C during this time due to the low capacity of the heating system in the auto-preparation plant for synthetic inorganic wastewater. During this period, the average VNR was 4.84 kg-N/m^3^/day. Table 3 shows that the average effluent NO_2_-N concentration was only 6.0 mg/L.

An unfavorable situation occurred, in which the influent NH_4_-N concentration was lower than the target value (Fig. 5a) because of improper measurement of the NH_4_-N concentration at low temperature. The average reactor temperature was 31.9 °C during Run IV and 29.6 °C during Run V (Table 1). During Run IV, the target temperature was 35 °C but the actual reactor temperature did not reach 35 °C due to the low heating capacity. Therefore, the target temperature was reduced from 35 to 30 °C during Run V but the actual decrease in the reactor temperature was only 2.3 °C, not 5 °C as expected. The average VNR in Run IV was 4.84 kg-N/m^3^/day (Table 4), while that in Run V was 4.80 kg-N/m^3^/day. This indicates that this level of temperature decrease did not have much influence on VNR. Figure 3 shows the reaction ratio during anammox treatment. The reaction ratios for NO_2_-N/NH_4_-N and NO_3_-N/NH_4_-N were calculated at 1.24 and 0.11, respectively, from the slopes of the regression lines.

**Table 4:** Comparison of wastewater characteristics and treatment results of anammox process

Item	Sludge reject water^a^	Synthetic wastewater^c^	Sludge reject water	Semiconductor factory wastewater	Municipal sludge reject water
Influent wastewater before pretreatment					
NH_4_-N (mg/L of N)	88–32	154–205	88–29	250–400	500–1,100^e^
NO_2_-N (mg/L of N)	**–**	196–237	**–**	**–**	**–**
TOC (mg/L of C)	17–689	0.5–2.5	28–645	Few	Unknown
pH (–)	7.2–8.3	7.1–7.6	6.5–8.0	Unknown	Unknown
SS (mg/L)	**–**	0	0–13,699	Few	Unknown
Anammox reactor					
Anammox reactor volume (L)	19.2	416.4	11.4	58,000	2.6
VNR (kg-N/m^3^/day)	8.78 (ave.)^b^	4.84 (ave.)	4.17 (max.)	2.0–3.0	2.95 (max.)
HRT (min)	60 (ave.)^b^	99 (ave.)	42	152^d^	240
Influent C/N (after pretreatment)	0.054 (ave.)^b^	0.003 (ave.)	0.196	0	Unknown
Reference	This study	This study	Okamoto et al. (2009a, b)	Tokutomi et al. (2007)	Kaneshiro et al. (2009)

^a^Calculated without abnormal value

^b^For Run 2

^c^For Run IV

^d^Calculated using data from reference

^e^Calculated using data from reference

## Discussion

**Fig. 6:**
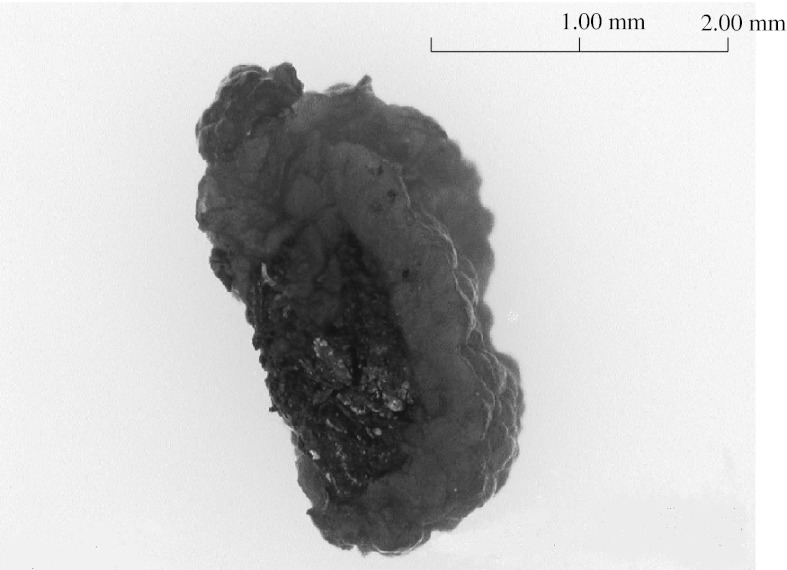
Micrograph of anammox biomass attached on a malt ceramics carrier particle

In our previous study with an 11 L anammox reactor using MCL as a biomass carrier, a maximum VNR of 4.17 kg-N/m^3^/day was obtained (Okamoto et al. 2009a, b). In the present study, MCP was used as the biomass carrier for anammox sludge in the both reactors as the main carrier and only a small quantity of MCL was used for the support of MCP in the 400 L anammox reactor. The VNR obtained with the 20 L anammox reactor was higher than that obtained for the 11 L anammox reactor. The specific surface area of MCP is larger than that of MCL and this increased the VNR for the 20 L anammox reactor. The high settling velocity of MCP covered by anammox sludge could also reduce the washout of sludge and enable the high VNR. Figure 6 shows a micrograph (VC7700, Omron Corp., Kyoto, Japan) of the red MCP covered by anammox sludge taken from 20 L anammox reactor on day 331. This photo shows that a high density of anammox sludge was tightly attached to the MCP. Table 4 shows a comparison of the results obtained with previous reports. Tokutomi et al. (2007) reported that the nitrogen VNR reached 2.0–3.0 kg-N/m^3^/day for the treatment of real wastewater with no organic compounds or SS from a semiconductor plant. The HRT in their report was calculated to be 152 min. In this study, the average VNR was greater than that reported by Tokutomi et al. and reached 8.78 kg-N/m^3^/day with a HRT of only 1 h (Table 4). In the experiment using synthetic wastewater for 86 days of operation (Run IV), an average VNR of 4.84 kg-N/m^3^/day was obtained with a HRT of 1.7 h. These results suggest that rapid nitrogen removal is possible with a plug flow anammox reactor using MC as biomass carriers. The filtrate from the sludge dehydrator used as influent for the 20 L reactor in the present study had a lower NH_4_-N concentration than the influent NH_4_-N concentration used in the previous study.

Treatment of wastewater with a low NH_4_-N concentration under a high VNR, requires the operation under a short HRT. The optimum temperature for anammox bacteria is reported to be around 30 °C (Strous et al. 1997) or 37 °C (Isaka 2008). In consideration of this, our results (Table 1) suggest that temperature control of the reactor is required to maintain the optimum reactor temperature. The calculated reaction ratios for NO_2_-N/NH_4_-N and NO_3_-N/NH_4_-N were 1.32 and 0.26, respectively, which are in agreement with the reaction ratios reported by Strous et al. (1998).

During operation with the 20 L anammox reactor, the influent contained a low concentration of TOC and only 10 % of the TOC was removed during anammox treatment (Fig. 4). This was also observed in our previous study (Okamoto et al. 2009a, b). The influent organic compounds might be utilized as electron donors in heterotrophic denitrification and may contribute to the reduction of NO_3_-N produced by the anammox reaction. Therefore, the actual NO_3_-N/NH_4_-N reaction ratio was lower than the reported ratio of 0.26 (Fig. 3). With the 400 L anammox reactor, the reaction ratio was also lower (Fig. 3) than that reported by Strous et al. (1998). However, synthetic inorganic wastewater was used in this case. Therefore, the above assumption would not be appropriate in this case. Further studies are required to clarify the reasons for the difference in reaction ratio.

As a result of bacterial community analysis (Table 2) for the 20 L anammox reactor, Asahi BRW1, which we first detected in previous study (Okamoto et al. 2010), was detected as the dominant anammox bacterium. On the other hand, from the sludge used to seed the 400 L reactor, anammox bacteria were detected as 40 of the 53 clones. In the previous study (Okamoto et al. 2010), Asahi BRW1 was found in the microbial community of the sludge in a partial-nitritation reactor treating filtrate from the sludge dehydrator in Asahi Breweries. Thus, the Asahi BRW1 should originate in the wastewater collected from Asahi Breweries.

## Conclusions

An anammox reactor using MC, which is a type charcoal made from spent grains, was developed and characterized. The average VNR reached 8.78 kg-N/m^3^/day in the 20 L anammox reactor treated brewery wastewater with 1 h of HRT. The average VNR stabilized at 4.84 kg-N/m^3^/day in the 400 L anammox reactor treating synthetic inorganic wastewater with 1.7 h of HRT. These results suggest that the MC reactor could be used to treat wastewater under short HRT. In the 20 L reactor, Asahi BRW1, probably originating from the wastewater collected at Asahi Breweries, was detected as the dominant anammox bacterium.
